# What hinders and helps academics to conduct Dissemination and Implementation (D&I) research in the field of nutrition and physical activity? An international perspective

**DOI:** 10.1186/s12966-020-0909-z

**Published:** 2020-01-16

**Authors:** Harriet Koorts, Patti-Jean Naylor, Rachel Laws, Penelope Love, Jaimie-Lee Maple, Femke van Nassau

**Affiliations:** 1 Institute for Physical Activity and Nutrition (IPAN), School of Exercise and Nutrition Sciences, Deakin University, Geelong, Australia; 2 School of Exercise Science, Physical and Health Education, University of Victoria, Victoria, BC Canada; 3 Department of Public and Occupational Health and Amsterdam Public Health research institute, Amsterdam UMC, Vrije Universiteit Amsterdam, Amsterdam, The Netherlands

**Keywords:** Implementation, Dissemination, Translation, Physical activity, Nutrition, Academia, Barriers, Facilitators, Real-world

## Abstract

**Background:**

Ineffective research-practice translation is a major challenge to population health improvement. This paper presents an international perspective on the barriers and facilitators associated with the uptake of and engagement in Dissemination and Implementation (D&I) research in the fields of physical activity and nutrition.

**Methods:**

A mixed methods study involving participants from the International Society for Behavioral Nutrition and Physical Activity (ISBNPA) network. Participants completed an online survey (May–July 2018) and/or participated in a focus group during the annual ISBNPA conference (June 2018). Descriptive statistics were generated for quantitative online and pre-focus group survey data. Fisher’s exact tests investigated associations of (i) length of time in academia, (ii) career stage and (iii) country of work, and agreement with ‘perceptions of D&I’. Qualitative data were analysed thematically.

**Results:**

In total, 141 participants responded to the survey (76% female, 21% aged 35–39 years, 14 countries represented) and 25 participated in focus groups (*n* = 3). Participants self-identified as having knowledge (48%), skills (53%) and experience supporting others (40%) to conduct D&I research. The majority (96%) perceived D&I was important, with 66% having organizational support for D&I, yet only 52% reported prioritizing D&I research. Perceptions of D&I differed by length of time in academia, career stage and country of work. Barriers included: (i) lack of D&I expertise; (ii) lack of organisational support/value for D&I; (iii) embedded scientific beliefs/culture; (iv) methodological challenges with D&I research; (v) funding/publishing priorities and; (vi) academic performance structures. Facilitators included: (i) increased presence/value of D&I; (ii) collective advocacy; (iii) organisational support for D&I; (iv) recruitment of D&I scientists and; (v) restructure of academic performance models, funding/publishing criteria.

**Conclusions:**

Individual, organisational and system-wide factors hindered academics’ engagement with and support for D&I research, which was perceived to reduce opportunities for research-practice translation. Factors were mostly consistent across countries and individual career stages/time spent in academia. Embedding D&I early within academic training, and system-wide reorientation of academic performance and funding structures to promote and facilitate D&I research, are some of the necessary actions to reduce the research-practice gap. Consistent with public health more broadly, these changes are long overdue in the fields of physical activity and nutrition.

## Background

Effective research-practice translation remains one of the major challenges to population health. The 17 year lag time between clinical evidence generation and practice implementation [1] has been well-documented, along with ongoing evidence for the substantial ‘research waste’ in medical research [2] (i.e., research that ignores the needs of target users and priorities of stakeholders, that is poorly designed and produces inaccessible and less translatable research outputs). These challenges are not limited to clinical fields; they exist across all disciplines of health research. More than a decade ago, Glasgow et al. identified fundamental methodological, funding and priority-orientated barriers to research translation in health promotion research, such as an overemphasis on linear, controlled trials over real-world effectiveness trials, and a limited consideration of the complexity of real-world practice compared to reductionist approaches [3]. Historically, as with many fields, physical activity and nutrition research has also been dominated by methodologies that may be less likely to inform real-world practice (e.g. Randomised Controlled Trials [RCT] designs), and physical activity intervention research often lacks planning for real-world implementation and translation [4].

In practice, evidence uptake and utilisation is confounded with an array of factors that can hinder effective implementation, which, without prior consideration or testing, can have substantial negative impact on research-practice translation. For example, intervention characteristics (e.g., adaptability), provider characteristics (e.g., skill proficiency at implementing), organisational factors (e.g., capacity to implement), and community-level factors (e.g., time and money for implementation) all influence effective implementation of evidence-based programs in practice [2].

Dissemination and Implementation (D&I) science seeks to address the research-practice gap, with the aim to understand ways of systematically facilitating implementation and utilization of evidence-based approaches to improve the quality and effectiveness of health promotion, health services, and health care [5]. D&I research has grown rapidly over the past few decades. Many models dedicated to D&I research have been identified [6, 7], and there are now multiple training institutes to support capacity building (e.g., the Training Institute for Dissemination and Implementation Research in Health [TIDIRH] [8]), and dedicated translational research funding schemes (e.g., The Australian National Health and Medical Research Council [NHMRC] Partnership Project grants) designed to increase collaboration between researchers and health-focused end-users to directly inform policy and practice [9]. Methodological advances and funding streams to support dissemination, implementation, and scale-up trials are promising; however, from discovery to delivery, effective translation of evidence-based programs and policies in practice are dependent on extensive interacting multilevel influences. Major principles of designing for dissemination include shifting funder priorities and processes (e.g., integrating dissemination planning as part of funding application requirements) and changing researcher incentives and opportunities (e.g., academic incentives, such as promotion, that are built around research impact) [10].

Increasingly, academics and academic institutions are required to demonstrate the real-world translatability and impact of their research. Maximising the transparency and reproducibility of physical activity and nutrition research [11] has been recognised as one way of improving research access. For example, in 2014, the UK introduced the Research Excellence Framework [12] as an evaluation system for assessing research impact and enlisting institutional accountability for research quality. Such systems provide incentive structures for academics and institutions to increase the demonstrable impact of research outputs. Impact evaluation systems can present a ‘carrot and stick’ approach to improving the mechanisms of *capturing* public health impact, and yet the challenges of research translation stem from the development of research priorities to begin with and the methodologies employed to answer them. Unsurprisingly, organisational factors, such as collective value placed on research translation and funding/academic incentive structures, therefore heavily influence the types of research pursued.

Despite calls for increased training of academics and practitioners in D&I research [10], and incorporation of concepts such as ‘research waste’ into research training and curricula [13], the extent that individual, organizational, and systemic factors within academia influence physical activity and nutrition research-practice translation remains unclear. Understanding what helps and hinders academics from conducting research that is more translatable into practice is critical to reducing the research-practice gap. The purpose of this study, therefore, was to gain an international perspective on barriers and facilitators associated with the conduct and uptake of D&I research within academia, specifically in the fields of physical activity and nutrition. Outcomes will contribute to identifying multilevel strategies required to facilitate, support and incentivise physical activity and nutrition research that aims to reduce the research-to-practice gap.

## Methods

A mixed method concurrent triangulation design [14] was used. This design type is characterised by the simultaneous collection and analysis of qualitative and quantitative data, which are integrated during interpretation phases [15]. The study involved an online survey and focus groups with members of the International Society for Behavioral Nutrition and Physical Activity (ISBNPA) network.

### Participants and recruitment

Participants were recruited prior to and during the ISBNPA Annual Congress held in Hong Kong, June 2018. Participants included members of the ISBNPA network (~ 1000), representing approximately 40 different countries (https://www.isbnpa.org/). For the purposes of this study, ‘members’ included those individuals who were registered with the ISBNPA society to receive ISBNPA communications (e.g., the online ISBNPA newsletter and/or social media) and delegates who attended the June 2018 conference. The ISBNPA network included academics, practitioners and stakeholders working in the fields of physical activity and nutrition, and participants were not required to have a paid society membership.

Online survey recruitment took place during May–July 2018 and focus group recruitment took place during May–June 2018. The online survey was sent to members of the ISBNPA network via a Qualtrics link distributed through bi-monthly e-newsletters sent by the ISBNPA communications team, and via the ISBNPA ‘Implementation and Scalability’ Special Interest Group (SIG) Twitter posts and e-newsletters over a 3-month period. Online survey participants were invited to take part in one of three focus groups during the 2018 ISBNPA conference, via a registration page on the final screen of the online survey. Recruitment for focus groups also took place during the conference via flyers and Twitter posts.

### Data collection

#### Online survey

Survey measures were generated based on the aims of this study to identify individual, organisational and system-wide constraints to conducting D&I research in academia, previously identified barriers to research translation [3], and researcher training needs in D&I [10] (Additional file 1). There were 11 questions in total, which included basic demographic information (7 questions), previous training in D&I (1 question), and perceptions of D&I research (1 question containing 17 items rated on a 5-point Likert scale). Perceptions of D&I research included assessment of individual level knowledge and engagement (4 items), perceived importance (1 item), skill level and self-efficacy to conduct this type of research (2 items), perceived value and impact (4 items), perceived relevance and applicability (2 items), and influencing factors at an organisational and systems level (4 items). Two open-ended survey questions identified participant views on major barriers and facilitators to enhancing research translation.

A pragmatic subsample of participants (*n* = 36) also participated in a test-retest study in May 2018 to evaluate the reliability of the survey items. Participants were recruited from three Universities in Australia, the Netherlands and Canada, and were asked to complete the online survey twice, with the second time up to 2 weeks from the first occasion. Test retest reliability of the 17 items (‘perceptions of D&I research’) was conducted using the original 5-point Likert scale for variables, and weighted Kappa using Stata’s default weighting matrix was used. Results showed that the 17 survey items (‘perceptions of D&I’) had fair to substantial test-retest reliability (Table 1), with Kappas ranging from between 0.20 and 0.75 (Mean Kappa = 0.50), all *p* values <.05.

**Table 1:** Perceived individual, organisational and system level factors related to engagement, uptake and support for D&I research in academia

	N^a^	Disagree (%)	Neither (%)	Agree (%)	Kappa^b^
Individual level factors					
D&I science is important to reduce research to practice gap	134	0.8	3.0	96.3	0.52
I have the skills necessary to conduct D&I research	134	19.4	27.6	53.0	0.71
I prioritize conducting/supporting D&I research (e.g. through supervision, provision of funding)	134	16.4	32.1	51.5	0.35
I have the knowledge required to conduct D&I research	134	23.1	29.1	47.8	0.75
I feel confident I could conduct D&I research	134	19.4	24.6	56.0	0.66
I have experience supporting others to engage in D&I research (e.g. through supervision, provision of funding)	134	41.8	18.7	39.6	0.62
I have experience conducting/being involved in (e.g. as a collaborator) D&I research	133	22.6	15.8	61.7	0.58
More often than not, I engage/collaborate with stakeholders and involve them in the design and conduct of my research	134	12.7	13.4	73.9	0.73
My research has real-world relevance	134	1.5	3.0	95.5	0.48
My research has real-world impact	133	2.3	9.0	88.7	0.43
I would like my research to have greater real-world impact	133	6.0	9.0	85.0	0.20
D&I science has the potential to improve the real-world impact of my research	133	3.0	9.0	88.0	0.57
D&I science is not immediately relevant/applicable to my area of research	133	88.0	3.8	8.3	0.46
Organisational level factors					
My supervisors/colleagues think it is important to conduct D&I research	134	9.0	20.9	70.2	0.51
My organisation supports me to conduct/engage in (e.g. as a collaborator) D&I research	133	9.0	24.8	66.2	0.32
System level factors					
Funding agencies in my country more likely to fund D&I research	133	17.3	37.6	45.1	0.40
Journals in field are less likely to publish D&I research	133	22.6	47.4	30.1	0.29

Data reported for ‘Disagree’ are a combined score relating to those who stated ‘Disagree’ and ‘Strongly disagree’. Data reported for ‘Agree’ are a combined score relating to those who stated ‘Agree’ and ‘Strongly agree’

*D&I* Dissemination and Implementation

^a^N’s differ due to questions not being completed in the surveys

^b^Test-retest reliability weighted Kappa from subsample (*n* = 36) only, all p values <.05

#### Focus groups

The semi-structured focus group questions explored participants’ level of understanding and engagement with D&I research, and the types of barriers and facilitators they experienced. Questions were framed around exploring differences across countries, disciplines and academic career stages (Additional file 2). Example questions included “*What would help you as an individual to conduct or support research that aims to reduce the research-to-practice gap*?” and “*What could the academic system (e.g. National Funding Agencies, peer-reviewed journals, and academic promotion structures) do to facilitate, support and incentivise research that aims to reduce the research-to-practice gap*?”. Focus groups were conducted by members of the research team experienced in running focus groups. Pre-focus group surveys captured basic demographic information about participants (e.g., gender, age, country of work), and were audio recorded (lasting approximately 30 min).

### Analysis

Descriptive statistics were reported for quantitative online and pre-focus group survey data. Continuous sample characteristics were presented as means and standard deviations (SD) and categorical data presented as counts and percentages. For overall sample descriptives, responses to the ‘perceptions of D&I’ question (containing 17 items) were combined into three groups classified as ‘agree’ (sum of responses ‘strongly agree’ and ‘agree’), ‘neither’ (response ‘neither agree nor disagree’) and ‘disagree’ (sum of responses ‘strongly disagree’ and ‘disagree’). Fisher’s exact tests were used to test for associations of (i) length of time in academia, (ii) career stage and (iii) country of work, and agreement with each of the 17 items (perceptions of D&I). For these tests, due to small cell sizes for the disagree and neither categories, these categories were combined into one category ‘disagree/neither’. Results for country of work were only reported for the five countries with the highest participation rate, since response rates for other countries were very small (i.e., *n* < 4). All analyses were conducted using StataSE 15 (StataCorp LP, College Station, Texas).

Qualitative data were transcribed verbatim and analysed thematically using NVivo 12. Thematic analysis involves initial familiarisation with the data, coding and tabulation of raw themes, which are grouped based on patterns of emergence and overlapping relevance [16]. Coding and theme development was firstly deductive, guided by the study aims and project team’s previous research and conceptualisation [17], followed by an inductive approach directed by the content of the transcripts [18]. A coding structure based on the aims of the study was developed by the research team prior to analysis. Thematic analysis was conducted by JLM, with a sample of coded transcripts checked and verified by HK. Instances of divergence from the predefined coding structure were discussed between JLM and HK until consensus was reached. Qualitative free text survey responses were coded thematically by FvN, checked and verified by the research team, and added to the focus group results.

## Results

### Quantitative survey data

In total, 141 participants (76% female, 21% aged 35–39 years old) completed baseline surveys, representing 14 countries (highest participation rate; Australia [39%], the Netherlands [19%], USA [11%], Canada [11%] and UK [10%]). Most participants (87%) were employed in an academic position (i.e., research, teaching and/or lecturing) and classified themselves as an early- or mid-career researcher (60%), and over half (53%) had worked or studied in an academic institution/University for < 10 years. The majority of participants worked primarily in the field of physical activity (65%) and public/population health (57%), followed by healthy diet/nutrition (37%), and only 12% of participants had previously undertaken formal D&I training.

### Perceived individual, organisational and system level factors associated with engagement, uptake and support for D&I research in academia

Table 1 presents perceived multilevel factors related to participants’ degree of engagement in, conduct of and support for D&I research as reported in the survey. At an individual level, the majority of participants agreed that their own research had real-world impact (89%) and this was something they would like to increase (85%). D&I science was perceived as being relevant and having the potential to improve the impact of participants’ research. Less than half of participants reported having neither the knowledge required to conduct D&I research or the experience supporting others to do so (i.e., through supervision), and approximately half perceived they had the necessary skills. Despite most participants agreeing that D&I science was important and that their own research had real-world relevance; only around half of the sample reported prioritizing conducting or supporting D&I research. At an organisational level, more than two thirds of participants agreed that their colleagues and supervisors thought that D&I research was important, yet only 66% thought that their organisation supported them to conduct or engage in D&I research. At a systems level, almost half of the participants (45%) reported that funding agencies were more likely to fund D&I research projects within their country than other types of research, whereas around a third perceived that journals were less likely to publish D&I research.

#### Differences by participant length of time in academia

Participants with > 20 years’ experience working in academia had a significantly greater than expected likelihood of having supported others to engage in D&I research (62%) (e.g., through supervision, provision of funding) compared to those participants with ≤; 10 years and > 20 years of experience (Additional file 3).

#### Differences by participant career stage

Participants > 10 years post PhD had greater than expected likelihood of having the knowledge to conduct D&I research (67%) and experience supporting others to engage in D&I research (e.g. through supervision, provision of funding) (73%), than academics (non-PhD), ECRs and MCRs (Additional file 4). Conversely, academics (non-PhD), had significantly greater than expected likelihood of reporting that D&I science was not immediately relevant or applicable to their area of research (27%), compared to ECRs, MCRs and those > 10 years post PhD.

#### Differences by participant country of work

There was overall agreement across individuals working in all countries that their research needed to have greater real-world impact. A significantly greater than expected likelihood of reporting this was observed for those working in Australia (91%) and the UK (100%) (Additional file 5). Participants working in the USA had a significantly greater than expected likelihood of reporting they perceived possessing the skills (94%), knowledge (75%) and confidence (88%) required to conduct D&I research, compared to those working in Australia, Canada, the Netherlands or the UK. Participants working in the USA also had a significantly greater than expected likelihood of prioritizing conducting or supporting D&I research (i.e., through supervision) (88%), experience conducting or being involved in D&I research (94%), and typically engaging/collaborating with stakeholders during the design and conduct of their research (94%). Those working in the Netherlands had lower than expected likelihood of having the skills (38%) and knowledge (33%) required to conduct D&I research, or to prioritizing conducting or supporting D&I research (38%), than those working in the USA, Australia or Canada. In comparison to these other countries, participants in Australia had lower than expected likelihood of having experience of (50%) and confidence conducting (43%) D&I research. Compared to the Netherlands (83%), USA (94%), Canada (93%) and UK (85%), only around half those working in Australia (56%) reported engaging and collaborating with stakeholders during the design and conduct of their research.

### Qualitative focus group data

Three focus groups involved 25 participants (76% female, 28% aged 45–49 years old), representing 11 countries (highest participation rate; Australia [24%], USA [16%], UK [12%], Denmark [12%] and Canada [8%]). All focus group participants worked in academia, 60% having worked in an academic institution/University for up to 10 years, and 44% were more than 10 years (full-time equivalent) post PhD. Most participants worked in physical activity (76%) followed by implementation/scale up (56%) research. Consistent with survey participants, 12% reported having previously undertaken formal D&I training. Below is a narrative of the key themes from focus groups, and Table 2 presents a summary of the major categories of barriers and facilitators corresponding to individual, organizational and system levels 2.

**Table 2:** Categories of barriers and facilitators to the uptake of, engagement in and support for D&I research in academia

Level	Barriers	Facilitators
Individual	**1. Insufficient D&I training/courses** ▪ Lack of knowledge, understanding and skills to conduct D&I research **2. Individual perceptions of scientific approaches** ▪ Perceived superiority of linear models of translation and preference for efficacy studies ▪ Perceived lack of ‘outcomes’ from D&I research; large ‘trade off’ and ‘lack of return on investment’ **3. Development and maintenance of stakeholder relationships** ▪ Funding and time required, and difficulties obtaining sufficient support/resources to conduct D&I research **4. Challenges of real-world research methodologies** ▪ Perceptions D&I is ‘messy’, difficult to design, interpret and write-up ▪ Perceived ‘intimidating’ field to enter into unskilled	**1. Increased opportunities for D&I in academia, research and teaching** ▪ Development of certified training/courses nationally, embedding D&I science within HDR teaching/training **2. Shifting cultural academic mindset and types of research** ▪ Managing academic expectations of D&I research, including increased acceptance of D&I high quality consensus methodologies **3. Partnership network developed a priori and over time** ▪ Maintenance of a ‘dynamic’ partner network across entire translation system; enhances flexibility during relationship/partner change **4. Leveraging off academic institution’s mission statement/objectives** ▪ Strategically aligning research and reinforcing academics’ responsibility for translation to increase perceived value **5. Collective advocacy to change the academic system** ▪ Academics collectively challenge journal ‘norms’ and culture of public health research to embrace a wider perspective of real-world impact
Organisational	**1. Lack of expertise in D&I science in academic institutions** ▪ Lack of trained D&I scientists limits capacity building in D&I **2. Embedded culture not conducive to translational research** ▪ Overemphasis on outcome-orientated metrics, top down approach of ‘pushing’ interventions onto communities ▪ Lack of understanding (internal and external) of the challenges conducting D&I research, underlying theory, validated methodology and measures; linked to perceived lack of value **3. ECR training (e.g., PhD programs) doesn’t facilitate D&I research** ▪ Lack of inclusion of D&I in HDR teaching/training; ECRs have minimal skills/knowledge of translation, reinforced via entrenched organisational practices ▪ Time required for stakeholder engagement exceeds PhD time/funding; difficult to build ECR capacity in D&I	**1. Increase employment/opportunities for D&I scientists** ▪ Targeted recruitment of trained D&I scientists, development of PhD programs dedicated to D&I science **2. Support for D&I involvement at different career stages** ▪ Incentivise and enable academic engagement in D&I science according to career stage demands, expectations and requirements **3. Shift organisational cultural towards research translation** ▪ Create organisational culture that values D&I and co-production of evidence with stakeholders **4. Collaborative knowledge sharing across institutions** ▪ Encourage and facilitate (e.g., resources/time) collaborations locally with stakeholders and across academic institutions
System	**1. Funding priorities and overemphasis on ‘innovation’** ▪ Short-term vision and priorities of funders/academic system not conducive to D&I science; reinforces perceived lack of value and priority of D&I and perceptions ‘less innovative’ **2. Demands for research impact vs. changes in funding environment** ▪ Increased academic pressure for demonstrable public health impact; funding agencies slow to support types of research required and lack of D&I expertise/understanding on funding review panels **3. Journal publishing criteria non-conducive to D&I research** ▪ Journal criterions counteractive to real-world/D&I research, less exposure of D&I research in major journals; creates uncertainty in publishing and thus disincentive for ECRs/academics **4. Outcome- and output-orientated academic performance structure** ▪ Reinforces perceived ‘un-appeal’ of D&I; dis-incentivises academics	**1. Funding and outcome metrics prioritising stakeholder involvement** ▪ Funding schemes dedicated to co-production/participatory approaches ▪ System incentives for collaborative working across organisations **2. Research translation embedded in academic performance metrics** ▪ Part of internal (e.g., University) and external (e.g., national assessments) reporting/assessment criteria **3. Restructure of academic system via Government leadership** ▪ Top-down pressure to enact system change and leverage country-wide resources for D&I; ‘upstream’ academic drive for change ‘piecemeal’ **4. Increase presence of D&I at national/international conferences** ▪ Via interest groups and networks of expertise within and associated with major conferences/societies **5. Journals dedicated to publishing D&I research** ▪ Increase journals/broadening selection criteria to facilitate and incentivise publishing of D&I research

Primary categories (numbered, bold) and subcategories generated from thematic analysis of three focus groups (*n* = 25 participants)

*D&I* Dissemination and Implementation, *HDR* Higher Degree Research, *RCT* Randomised Controlled Trial, *ECR* Early Career Researcher, *ISBNPA SIG* International Society for Behavioral Nutrition and Physical Activity Special Interest Group

### Barriers and facilitators to the uptake of, engagement in and support for D&I research in academia

Major barriers included the lack of D&I knowledge and training, historical linear approaches towards evidence generation, resources/funding required and methodological challenges involved in conducting real-world research. Facilitators related to the increased exposure of D&I research at national and international conferences, and shifting the academic culture and perceived norms regarding approaches towards generating evidence. Entrenched academic, system-wide, beliefs continued to overemphasize and enable studies focusing on internal validity.

#### Theme 1: embedded academic culture

Participants described the embedded culture of evidence generation and funding in academia as inappropriate and outdated for effective translation. Implementation was regarded as an afterthought for most academics and funders. This was referred to as ‘research waste’:

*“Well I work at a university in the United States that’s a land grant university…they’re supposed to be taking research…and implement that and share it with society. But we’ve been doing that for…a hundred years or something, and there’s not really a scientific aspect to that…it seems like how much money are we going to continue to waste and not do this in a more rigorous and academic space?”* [Respondent 1, Focus Group 2]

“*When we’re looking for programs to roll out or to scale up there’s actually very few in the literature that are suitable, and so there’s a lot of research waste. There’s a considerable amount of investment in interventions that aren’t particularly scalable*.” [Respondent 4, Focus Group 1]

Limited organisational support for D&I conduct and a culture perceived to be non-conducive to practice-based research (e.g., that prioritized controlled trials and demonstrable short-term efficacy), meant real-world implementation lacked priority and understanding:

“*I think they* [the organisation] *don’t quite get that, you know, there might be an effective program or policy or whatever, they* [the organisation] *don’t perhaps really get that we’re trying to help people implement that. It’s probably the thought that if you’ve got something effective it’s just going to be up taken…so I think that maybe it’s just* [the organization’s] *lack of experience of working in that area*…” [Respondent 3, Focus Group 3]

Facilitators included shifting organisational cultures and norms, such as embedding implementation planning from the outset in projects, and increasing support for co-production and participatory research within the community as routine research practice. Participants stated that translation should not be considered a ‘separate’ component to the research process, and the responsibility for co-production was both internal and external to Universities:

*“I think, you know, researchers need to learn to be involved in co-designing programs, and practitioners you know need to be invited into co-design research. So there’s, you know there’s those things from both sides*.” [Respondent 2, Focus Group 3]

#### Theme 2: trade off

In particular, for ECRs, there was the perception among academics with supervisory responsibilities that a significant trade off existed and ‘lack of return on investment’ when conducting D&I research:

*“The real issue is…I think looking at it from academia, that it is a lot of work that you do not necessarily get any capital of, meaning publications. So our return on investment, when we invest in D&I, is tricky. And that is especially for instance an issue with younger researchers, because is it all right that I send them down a path where they spend all their time on stuff they won’t get in any publications?”* [Respondent 6, Focus Group 2]

Participants identified that often the ‘traditional’ research designs weren’t applicable to real-life scenarios, despite being valued within academia and among academics. The realities of real-world environments subsequently hindered their ability to conduct research traditionally considered as ‘best practice’. Whilst D&I was appropriate to study real-world implementation, it was perceived as less ‘valued’ within the broader academic community and seen as a ‘soft’ science:

*I think there is some barriers to researchers like us, that people will just say that what we do is program evaluation, that it’s not necessarily research, you’re just evaluating programs. And so that’s a bit of a barrier on the academic side* [Respondent 2, Focus Group 2]

Organisational support for engaging in D&I science at different career stages was mentioned as a key facilitator. Participants referred to this in context of reducing the disadvantage against ECRs wishing to purse this type of research, whilst increasing the value of it among more senior academics to support their career trajectory. Senior academics can ‘absorb’ the trade-off of this type of research:

*“how you can support that* [D&I research/stakeholder engagement] *being done at different stages in people’s career…if you’re less dependent on that* [outputs] *for promotion, then there’s more obligation to try and write those* [papers/grants], *of course including co-authors who are at an early stage of their career. But I think it’s a real dilemma*.” [Respondent 3, Focus Group 2]

#### Theme 3: ‘messy’ science

Perceptions that D&I science was ‘messy’ and complex to conduct compared to controlled trials, and the researcher’s desire to control implementation, meant this research was considered as intimidating and less desirable as a discipline:

*“I think it’s more difficult to, you know it’s clearer, it’s more structured, it’s easier, more like a recipe, you know, the standard way of doing it. What we’re moving into is more handwork in a way, you really have to be good in your stuff if you want to pull it together.”* [Respondent 3, Focus Group 1]

Participants expressed difficulties publishing D&I research due to journal criteria that is less likely to allow for real-world data and implementation designs, and how this impacted their perceptions of the field:

“*The issue in terms of publishing it, is we usually have these big, messy studies…and if we’re trying to publish them as a whole in one paper, it’s too complex…there’s just the messiness that comes from doing this sort of thing in the field almost means you just can’t satisfy sort of journals and the standards they hold themselves to*.” [Respondent 2, Focus Group 3]

*“If you take a PhD* [it’s] *still easier to carve out four Papers that we’re sure are spot on for Journals that are dealing with efficacy, and dealing with those kind of issues. It’s much more easier to handle* [than implementation research]. [Respondent 6, Focus Group 2]

#### Theme 4: capacity and skill building

Lack of institutional expertise, support and capacity for D&I science, conflicting cultural norms and inadequacies of Postgraduate training programs were major barriers. There was consensus regarding the lack of trained implementation scientists in the field, implementation experts to reach out to and lack of inclusion of D&I within Undergraduate and Postgraduate courses:

*“when I did my graduate degree there was no talk about implementation sciences…never was the focus in the lectures on moving past the RCTs. It was that was the golden standard. There was no talk about taking the research and applying it into the real world.”* [Respondent 8, Focus Group 2]

#### Theme 5: flawed academic system

At a systems-level, research funding structures, publishing criteria and academic performance indicators were the most frequently reported systemic barriers to research translation. This was endorsed by the survey data. Not only were these systems described as inhibiting the conduct of D&I research more broadly in academia, but also dis-incentivizing students and discouraging ECRs from entering the field to begin with.

*“Well I was quite discouraged to, when I came in to actually do a PhD to engage with stakeholders, and I was quite surprised by that”* [Respondent 4, Focus Group 3]

*“…what’s intimidating to me is this research results driven academic road that I may or may not step in…there’s an intimidation factor I think in some sense of starting in implementation sciences for that reason.”* [Respondent 8, Focus Group 2]

Academic systems subsequently reinforced existing negative perceptions about engaging in D&I science and thus ‘damaged’ the profile of D&I within the broader scientific community:

“*But I think there is a real dilemma. I think it might be possible to write the odd three or four star Paper to be included within the REF* [Research Excellence Framework]…[but] *I think for an early career scientist, unless they could get a three or four star Paper around that, unless they’re very prolific, then it’s potentially career suicide* [to pursue D&I science].” [Respondent 3, Focus Group 2]

Overemphasis among stakeholders and funders for innovation and impact negatively influences researchers desire to conduct D&I research:

“…*they’re* [stakeholders/funders] *explicitly only interested in innovation and not on actually being able to sort of take this* [intervention/evidence] *to another, you know another level*.” [Respondent 3, Focus Group 2]

This was described in context of a ‘flawed system’ lacking commitment to long-term outcomes and funders’ lack of recognition for the importance of practice or policy impact:

*“I think the entire system is working here against us, because the entire system is not designed to support studies which would follow up”* [Respondent 6, Focus Group 3]

“*Often we are funded for very short term impact, and you know it’s quite costly to look at the long term impact…which is really where most of us are wanting to see the data. So I would agree that short vision in terms of funding does really affect our implementation*.” [Respondent 4, Focus Group 3]

Conversely, one participant described that the increasing push for translating research into practice among academics, whilst having a lack of understanding about D&I, had led to unintended dissemination efforts driven by such external incentive:

“*We’ve noticed…because there’s so much money around implementation…academics are pushing the dissemination of trials that don’t even work... So it’s kind of like an unintended consequence of that push at the moment of translation*.” [Respondent 4, Focus Group 1]

System facilitators centered on restructuring academic performance benchmarks and a need for ‘top-down’ government leadership to enact change:

*“…you have to build a system where it makes sense to do stuff like this* [research translation], *because academics are, you know, fairly intelligent, so they know which way to go when it comes to performing well.”* [Respondent 6, Focus Group 2]

*“In my opinion there is need for more system change, because I mean or else all of us will be spending our time in communities, which will be a lot of fun, but you know fighting uphill* [to change the system].” [Respondent 6, Focus Group 2]

#### Theme 6: exposure of D&I research

Despite acknowledgement that a focus on translation and implementation was increasing in the field, the overall lack of journals publishing research in this space led to uncertainty over academic outputs and was detrimental to the exposure and precedence of D&I research:

*“But for me that* [publication bias] *tends to sort of ghettoise the research…you get these Journals that have buckets of qualitative research…they’re not in the mainstream, they’re not being read in say the BMJ or the Lancet, or you know they’re sort of Journals where people are going to find out what’s happening.”* [Respondent 2, Focus Group 3]

#### Theme 7: collective advocacy and incentivization

Participants expressed that when there are incentives to work collectively as researchers, D&I is more achievable. Academics perceived an individual responsibility to strive for change as part of collective advocacy:

“…*if we as a public health… community are able to kind of challenge those things collectively, rather than individually, then maybe we can actually shift the editorial policy a bit faster*.” [Respondent 3, Focus Group 2]

“*I think it should be throughout public health and health care, academia, all of us. We should be driving this forward*.” [Respondent 1, Focus Group 2]

A ‘carrot and stick’ approach to incentivising Universities and academics was also discussed, in terms of external assessment frameworks requiring demonstrable institution impact and new funding schemes dedicated to translational research:

*“We’re all obsessed, because we have to be, with the REF* [Research Excellence Framework]…*and therefore the flow of funding for research to Universities, so no University in the U.K. can afford to ignore it at the moment.”* [Respondent 3, Focus Group 2]

*“…we have the MRFF* [Medical Research Future Fund]…*they don’t want new ideas, they don’t want efficacy, you have to be able to take a project in 12 months and scale it…that will force academics to work into implementation…so then your organisation has to support you to do that”* [Respondent 7, Focus Group 2]

## Discussion

To our knowledge, this in the first study to obtain an international perspective on the multilevel barriers and facilitators to the uptake of, engagement in and support for D&I research among academics working in the physical activity and nutrition field. Sixteen years ago, Glasgow et al. (2003) highlighted that methodological, funding and priority-orientated barriers hindered research-practice translation, concluding that ‘*To produce significant improvement in the current state of affairs, changes will be necessary on the part of researchers, funding organizations, journal reviewers, and grant review panels’* [3]; and this study demonstrates that such challenges remain pervasive within academia.

There was a strong consensus among participants that D&I research was important to reducing the research-practice gap with participants wanting to increase the real-world impact of their own research. A lack of D&I training and expertise, entrenched beliefs and culture regarding how to generate evidence and achieve real-world impact, and practical challenges (e.g., time, costs, partnerships) associated with D&I research hindered individuals’ involvement. When comparing perceptions of D&I by country, US based researchers reported consistently higher levels of knowledge, skills and confidence in conducting D&I research. US based researchers also had greater perceptions that D&I research was a priority and that they had the experience engaging with stakeholders during the design and conduct of their research. This is potentially unsurprising given the number of training schemes available to support D&I research in the USA compared to other countries, and a long history of USA funding schemes to support research translation through the National Institutes of Health (NIH) [19]. Nonetheless, consistent with this study, the importance of, and need for, increased expertise and training in D&I science, and research-practice translation more broadly across the health disciplines, has been well-documented [10, 19–21]. Skill and knowledge development is an integral part of capacity building in D&I research, in particular among junior and pre-doctoral students. With around only half of all participants in this study indicating that they had the knowledge and skills required to conduct D&I research, increased training and capacity building appears essential for the field across all levels of the academic spectrum (i.e., Undergraduate training through to senior management).

Although evidence for increased skill and knowledge for D&I in some countries is promising, far fewer participants actively prioritized conducting or supporting others to engage in D&I research, or perceived that their organisation supported them to conduct or engage in D&I research. Experience mattered, however; those with more than 10 years post PhD reported supporting others to engage in D&I research through supervision or provision of funding. This is to be expected given the time commitment and network of stakeholders typically involved in D&I research, both of which are more likely achieved over the course of an academic career. The overall proportion of senior academics (62%) reporting this remained modest, nonetheless, and importantly, irrespective of country of work. The multilevel barriers to engaging in and supporting D&I research were broadly consistent across all participants. The lack of organisational understanding, support and perceived value of D&I science was consistently described among participants, suggesting that training may need to target not only individual-level skills and knowledge, but also individual and organisational culture and climate for this type of research. Previously, inadequate infrastructure and support systems have been identified as major barriers to research translation [3]. Without changes to the academic culture (norms and practices), at both organizational and systems levels, solely increasing the provision of D&I training may be insufficient.

More recently, systemic changes to some academic funding and metric systems have occurred. These are demonstrated by changes to grant requirements and additional funding streams globally, and by the expansion of courses and training programs to support D&I science. Canada launched the Population Health Intervention Research (PHIR) initiative [22], which is a strategic alliance bringing together funders, non-government agencies, policy-makers and researchers to increase integration of population and public health evidence into everyday practice. More recently, in 2018, Ireland launched their first TIDIRH [23], and in 2018, one of Australia’s national funding agencies (the Australian Research Council [ARC]) introduced a national assessment of the ‘engagement and impact’ of University research, to measure academic engagement with end-users and institutional research translation into, for example, economic, social and environmental gain [24]. Such progress and development is promising and, undisputedly, should be encouraged as the difficulty and time required to change existing and embedded systems of practice cannot be understated.

Nonetheless, despite global differences in funding structures, cultures and ways of conducting research, the gap between evidence generation and application remains across disciplines. Somewhat promising is that strategies to enhance D&I uptake and research-practice translation are potentially therefore generalizable internationally in the physical activity and nutrition fields. Nonetheless, the perception which emerged in this study that D&I research provided a ‘*lack of return on investment*’ and was ‘*career suicide*’ undermines current progress and is concerning for a number of reasons. Firstly, it reflects the misplaced system-orientated conceptualization of ‘impact’ based on academic-driven outcomes as opposed to real-world benefit, and thus the underlying motivations driving academic pursuits that generate scientific knowledge. Secondly, it reemphasizes the vast disconnect between the core purpose of public health research (i.e., to improve the health and lives of those in the community) and the entrenched beliefs regarding success and outcomes within academic environments.

To reorientate the values and priority placed on engaging in and supporting D&I research within academia, vital changes are required at an individual, organisational and systems level. At a systems level, for example, to build adequate research capacity, enablers are required within both funding and institutional environments [19]. More investment into reorienting the academic system and increasing the value, priority and opportunities to conduct physical activity and nutrition research within the paradigm of ‘real-world impact” is required. To help accelerate research-practice translation, strategies could include, but are not limited to, for example: (i) increased prioritization among funders for stakeholder co-designed long-term research, to ensure not only practice and policy relevance, but that findings are implementable, and implementation and scale up is taken into account during development of interventions; (ii) a restructure of academic performance metrics, both within and external to academic institutions (e.g., University promotion models, tenure tracks) to prioritise and demonstrate value of translational research impacts and; (iii) diversification of institutional recruitment models to encourage the hiring and promotion of academics based on ‘outputs’ broader than primarily traditional metrics (e.g., number of publications and grants); (iv) increased employment opportunities that enable ‘joint-appointments’ between academia and practice/policy to facilitate embedded research; and (v) training schemes to encourage practitioners and policymakers to undertake PhDs’ and postdoctoral fellowships within their organisations to build research into daily practice and practice into research.

The inclusion of a diverse range of academics in this study, based on career stage, experience with D&I and country of work, is a key strength of this study. Likewise, the use of mixed methods enabled a deeper understanding of barriers and facilitators to engagement in D&I science, and research which is potentially more translatable into practice. This study is, however, not without limitations. Firstly, the majority of participants (98%) represented high-income countries. Whilst our recommended strategies to enhance research-practice translation are potentially generalizable internationally given the consistency in results regarding participants’ experiences of engaging in D&I science, it is unknown how generalizable these findings are to low and middle income countries. Secondly, recruitment for focus groups specifically targeted all members of the ISBNPA network, however, for feasibility purposes, the focus group sessions were conducted during the ISBNPA ‘Implementation and Scalability’ special interest group meeting. Given that those individuals specifically interested in dissemination, implementation and scalability typically attend this meeting, there is the potential that focus group participants overrepresented those with an interest in D&I science. Participants may have therefore had greater exposure to the barriers to D&I research compared to other physical activity and nutrition researchers. Nonetheless, the barriers and facilitators identified from the online survey and focus groups were consistent, thus strengthening the conclusions of this study.

## Conclusions

Individual, organisational and system-wide factors hindered academics’ engagement with and support for D&I research, which was perceived to reduce opportunities for research-practice translation. Factors were mostly consistent across countries and individual career stages/time spent in academia. Embedding D&I early within academic training, and system-wide reorientation of academic performance and funding structures to promote and facilitate D&I research are some of the necessary actions to help reduce the research-practice gap. As with public health more broadly, these changes are long overdue in the fields of physical activity and nutrition.

## Supplementary information

**Additional file 1.** Online survey questions.

**Additional file 2.** Focus group questions.

**Additional file 3.** Levels of perceived understanding, knowledge, engagement with and importance of D&I by length of time in academia, Table comparing differences in perceptions of D&I by participant length of time in academia.

**Additional file 4.** Levels of perceived understanding, knowledge, engagement with and importance of D&I by academic career stage, Table comparing differences in perceptions of D&I by participant career stage.

**Additional file 5.** Levels of perceived understanding, knowledge, engagement with and importance of D&I by country of work, Table comparing differences in perceptions of D&I by participant country of work.

## Data Availability

The datasets used and/or analyzed during the current study are available from the first author on reasonable request.

## Abbreviations

D&I: Dissemination and Implementation
ECR: Early Career Research
FTE: Full time equivalent
HDR: Higher Degree Research
ISBNPA: International Society for Behavioral Nutrition and Physical Activity
RCT: Randomised Controlled Trial
SIG: Special Interest Group
