# Supplementary material for: What hinders and helps academics to conduct Dissemination and Implementation (D&I) research in the field of nutrition and physical activity? An international perspective
Source: Int J Behav Nutr Phys Act. 2020 Jan 16;17:7. doi: 10.1186/s12966-020-0909-z (PMC6966833; doi:10.1186/s12966-020-0909-z)
Supplement: Supplementary file 1 — Additional file 1. Online survey questions. [file 12966_2020_909_MOESM1_ESM.docx]

**Additional File 1: Online Survey**

| **About You** |
| --- |

1. **In which country do you primarily work? (Please note, if you regularly work in more than one country, please select the country where you spend the majority of your time)**

*DROP DOWN LIST OF COUNTRIES*

1. **Which of the following best describes your gender?**

| Male |  |
| --- | --- |
| Female |  |
| Other |  |
| I prefer not to say |  |

1. **Please select your age range**

| 18-24 years |  |
| --- | --- |
| 25-29 years |  |
| 30-34 years |  |
| 35-39 years |  |
| 40-44 years |  |
| 45-49 years |  |
| 50-54 years |  |
| 55-59 years |  |
| 60+ years |  |

1. **In which sector do you currently work/study?** **(Please select all that apply)**

| University/academic institution |  |
| --- | --- |
| Research consultancy |  |
| Federal government |  |
| State or local government |  |
| Non-government organisation (NGO) |  |
| Not-for-profit organisation (e.g. religious, charity organisation) |  |
| Community-based organisation |  |
| Other |  |

**4a. POP UP IF SELECT ‘OTHER’, ASK THEM TO SPECIFY**

1. **How long have you been working/studying in this sector?**

🞏_1_ Less than one year

🞏_2_ 1-5 years

🞏_3_ 6-10 years

🞏_4_ 11-15 years

🞏_5_ 16-20 years

🞏_6_ 21-25 years

🞏_7_ Over 25 years

1. **In which area(s) do you primarily work or study?** **(Please select all that apply)**

| Physical activity |  |
| --- | --- |
| Sedentary behaviour |  |
| Healthy diet/nutrition |  |
| Public health/population health |  |
| Clinical health |  |
| Sports medicine |  |
| Nutrition/physical activity policy |  |
| Implementation/scale up |  |
| Psychology |  |
| Sociology |  |
| Epidemiology |  |
| Health Services Research |  |
| Other |  |

**6a. POP UP IF SELECT ‘OTHER’, ASK THEM TO SPECIFY**

1. **What is your current position? (Please select all that apply)**

| Academic (Research, teaching and/or lecturing) |  |
| --- | --- |
| Industry professional |  |
| Practitioner/clinician |  |
| Consultant |  |
| Policy maker/policy officer |  |
| Senior manager/CEO of organisation |  |
| Student (including higher degree/research) |  |
| Other |  |

**7a. POP UP IF SELECT ‘OTHER’, ASK THEM TO SPECIFY**

**7b. POP UP if select option ‘Academic (Research, teaching and/or lecturing)’ (Qu 9)**

**What is your current career stage?**

| Academic (non- PhD) |  |
| --- | --- |
| Early Career Researcher (<5 years full-time equivalent post PhD) |  |
| Mid-Career Researcher (5-10 years full-time equivalent post PhD) |  |
| More than 10 years full-time equivalent post PhD |  |
| Other |  |

**7c. POP UP IF SELECT ‘OTHER’, ASK THEM TO SPECIFY**

| **Dissemination and implementation (D&I) research** |
| --- |

*The aim of Dissemination and Implementation (D&I) science/research is to understand how to systematically bring evidence-based policies and programs into real-world practice to promote health and prevent disease.*

*Other terms you may be aware of include: Implementation Science, Knowledge Translation, Translational Research, and Implementation Research.*

1. **Have you undertaken any formal training in D&I science?** (e.g. the Training Institute for Dissemination and Implementation Research in Health [TIDIRH], the Mentored Training for Dissemination & Implementation Research in Cancer [MT-DIRC], an in-person workshop/online course, or as a core component/major within a Degree).

| Yes |  |
| --- | --- |
| No |  |

**8a. POP UP IF SELECT ‘YES’, ASK THEM TO SPECIFY**

1. **Think about your own RESEARCH, the ACADEMIC SYSTEM in general and the ORGANISATION(S) you work for, how much do you agree with the following statements?**

|  | **Strongly disagree** | **Disagree** | **Neither agree nor disagree** | **Agree** | **Strongly agree** |
| --- | --- | --- | --- | --- | --- |
| D&I science is important to reduce the research to practice gap in physical activity and nutrition |  |  |  |  |  |
| I have the skills necessary to conduct D&I research |  |  |  |  |  |
| I prioritise conducting or supporting D&I research (e.g. through supervision, provision of funding) |  |  |  |  |  |
| My supervisor/colleagues think it is important to conduct D&I research |  |  |  |  |  |
| I have the knowledge required to conduct D&I research |  |  |  |  |  |
| My research has real-world relevance |  |  |  |  |  |
| More often than not, I engage/collaborate with stakeholders (e.g. end users) and involve them in the design and conduct of my research |  |  |  |  |  |
| I have experience supporting others to engage in D&I research (e.g. through supervision, provision of funding) |  |  |  |  |  |
| I feel confident I could conduct D&I research |  |  |  |  |  |
| My organisation supports me to conduct or engage in (e.g. as a collaborator) D&I research |  |  |  |  |  |
| My research has a real-world impact |  |  |  |  |  |
| I have experience conducting or being involved (e.g. as a collaborator) in D&I research |  |  |  |  |  |
| Journals in my field are less likely to publish D&I research |  |  |  |  |  |
| D&I science is not immediately relevant or applicable to my area of research |  |  |  |  |  |
| I would like my research to have a greater real-world impact |  |  |  |  |  |
| D&I science has the potential to improve the real-world impact of my research |  |  |  |  |  |
| Funding agencies in my country are more likely to fund D&I research |  |  |  |  |  |

1. **Think about academia in general. What do you think are the MAIN BARRIERS to conducting D&I science and research which has real-world translatability? (Please list as many examples as you can and provide an explanation where needed)**

**Barriers might include those you experience as an individual, within your organisation(s), and within academia more broadly.**

|  |
| --- |
|  |
|  |
|  |
|  |

1. **What is needed to improve the UPTAKE AND SUPPORT for D&I science in academia, to accelerate the translation of evidence in practice and policy settings? (Please list as many examples as you can and provide an explanation where needed).**

**Think about potential solutions for you as an individual, within your organisation(s), and within academia more broadly.**

|  |
| --- |
|  |
|  |
|  |
|  |
