# Supplementary material for: What hinders and helps academics to conduct Dissemination and Implementation (D&I) research in the field of nutrition and physical activity? An international perspective
Source: Int J Behav Nutr Phys Act. 2020 Jan 16;17:7. doi: 10.1186/s12966-020-0909-z (PMC6966833; doi:10.1186/s12966-020-0909-z)
Supplement: Supplementary file 2 — Additional file 2. Focus group questions. [file 12966_2020_909_MOESM2_ESM.docx]

**Additional File 2: Focus Group Schedule**

| **Domain** | **Topic and Probes** |
| --- | --- |
| **Levels of understanding, engagement with and perceived importance of conducting D&I research** | - What comes to mind when you think about D&I research? - Do you have experience with conducting, or supporting D&I research (i.e. through direct funding, supervision or collaboration on a project)? - Is D&I research something you would support / think is relevant / important? Why? Why not? (please ask both: why and why not) |
| **Types of barriers to conducting D&I research, exploring differences across countries, between disciplines and academic career stages;** | Suppose you or your colleagues **want to** conduct, fund or support D&I research   - What are **barriers** that you or your colleagues face? What makes it difficult? Why? (ask for explanation) - Are you aware of any differences across countries? - Do academics at different career stages experience different challenges?   *Carefully repeat back all factors cited by the respondents and ask if there are more* |
| **Individual (researcher) and organisational (internal and external institutional) strategies to facilitate, support and incentivise research that aims to reduce the research-to-practice gap.** | We are trying to identify **strategies to facilitate, support and/or incentivise research** that aims to reduce the research-to-practice gap.   - What would help **you as an individual** to conduct or support research that aims to reduce the research-to-practice gap? What is needed? Why? (ask for explanation)   If not covered, prompt for:   - Knowledge, perceived importance, skills, self-efficacy researchers - Institutional/academic system value/priority - Time - Funding - Influence on politics / health agenda - Communicate research findings - journals - Stakeholder partnerships - What could **your organisation** do facilitate, support and incentivise research that aims to reduce the research-to-practice gap? What is needed? Why? (ask for explanation) - What could **the academic system** (e.g. National Funding Agencies, peer-review Journals, academic promotion structures) do to facilitate, support and incentivise research that aims to reduce the research-to-practice gap? What is needed? Why? (ask for explanation)   *Carefully repeat back all factors cited by the respondents (+ and -) , and ask if there are more* |
| **Closing** | Is there anything else you think is important to mention that we have not talked about?   - Summarise - Thank respondents - Explain future steps of research project |
