# Supplementary material for: What hinders and helps academics to conduct Dissemination and Implementation (D&I) research in the field of nutrition and physical activity? An international perspective
Source: Int J Behav Nutr Phys Act. 2020 Jan 16;17:7. doi: 10.1186/s12966-020-0909-z (PMC6966833; doi:10.1186/s12966-020-0909-z)
Supplement: Supplementary file 3 — Additional file 3. Levels of perceived understanding, knowledge, engagement with and importance of D&I by length of time in academia, Table comparing differences in perceptions of D&I by participant length of time in academia. [file 12966_2020_909_MOESM3_ESM.docx]

**Additional File 3. Levels of perceived understanding, knowledge, engagement with and importance of D&I by length of time in academia**

|  | **Length time academia (% agreement)** | | | ***P*** |
| --- | --- | --- | --- | --- |
|  | <10 yrs  n (%) | >10 yrs < 20 yrs  n (%) | >20 yrs  n (%) |  |
| ***Individual level factors*** | | | | |
| D&I science is important to reduce research to practice gap | 66 (94.3) | 39 (97.5) | 21 (100.0) | 0.57 |
| I have the skills necessary to conduct D&I research | 34 (48.6) | 19 (47.5) | 15 (71.4) | 0.16 |
| I prioritize conducting/supporting D&I research (e.g. through supervision, provision of funding) | 30 (42.9) | 26 (65.0) | 10 (47.6) | 0.08 |
| I have the knowledge required to conduct D&I research | 27 (38.6) | 21 (52.5) | 13 (61.0) | 0.11 |
| I feel confident I could conduct D&I research | 34 (48.6) | 23 (57.5) | 15 (71.4) | 0.19 |
| I have experience supporting others to engage in D&I research (e.g. through supervision, provision of funding) | 15 (21.4) | 22 (55.0) | 13 (61.9) | **0.001** |
| I have experience conducting/being involved in (e.g. as a collaborator) D&I research | 37 (53.6) | 28 (70.0) | 14 (66.7) | 0.21 |
| More often than not, I engage/collaborate with stakeholders and involve them in the design and conduct of my research | 51 (72.9) | 29 (72.5) | 16 (76.2) | 1.00 |
| My research has real-world relevance | 66 (94.3) | 38 (95.0) | 21 (100.0) | 0.73 |
| My research has real-world impact | 61 (88.4) | 35 (87.5) | 19 (90.5) | 1.00 |
| I would like my research to have greater real-world impact | 59 (85.5) | 34 (85.0) | 17 (81.0) | 0.89 |
| D&I science has the potential to improve the real-world impact of my research | 59 (85.5) | 36 (90.0) | 19 (90.5) | 0.76 |
| D&I science is not immediately relevant/applicable to my area of research | 9 (13.0) | 2 (5.0) | 0 (0.0) | 0.13 |
| ***Organisational level factors*** | | | | |
| My supervisors/colleagues think it is important to conduct D&I research | 49 (70.0) | 29 (72.5) | 14 (66.7) | 0.87 |
| My organisation supports me to conduct/engage in (e.g. as a collaborator) D&I research | 44 (63.8) | 28 (70.0) | 13 (61.9) | 0.78 |
| ***System level factors*** | | | | |
| Funding agencies in my country more likely to fund D&I research | 30 (43.5) | 17 (42.5) | 11 (52.4) | 0.76 |
| Journals in field are less likely to publish D&I research | 17 (24.6) | 11 (27.5) | 11 (52.4) | 0.06 |

Data reported is a combined score relating to those who stated ‘Agree’ and ‘Strongly agree’. ECR – Early Career Researcher, MCR – Mid-Career Researcher, FTE – Full Time Equivalent, D&I – Dissemination and Implementation. Fisher’s exact tests were used to test for associations between length of time in academia and agreement with each of the 17 items (‘perceptions of D&I’); p value significant at <0.05 (bold).
