# Supplementary material for: What hinders and helps academics to conduct Dissemination and Implementation (D&I) research in the field of nutrition and physical activity? An international perspective
Source: Int J Behav Nutr Phys Act. 2020 Jan 16;17:7. doi: 10.1186/s12966-020-0909-z (PMC6966833; doi:10.1186/s12966-020-0909-z)
Supplement: Supplementary file 4 — Additional file 4. Levels of perceived understanding, knowledge, engagement with and importance of D&I by academic career stage, Table comparing differences in perceptions of D&I by participant career stage. [file 12966_2020_909_MOESM4_ESM.docx]

**Additional File 4. Levels of perceived understanding, knowledge, engagement with and importance of D&I by academic career stage**

|  | **Career stage (% agreement)** | | | | ***P*** |
| --- | --- | --- | --- | --- | --- |
|  | Academic (Non-PhD)  n (%) | ECR (<5yrs FTE post-Phd)  n (%) | MCR (5-10yrs FTE post-PhD)  n (%) | >10 years FTE post-PhD  n (%) |  |
| ***Individual level factors*** | | | | | |
| D&I science is important to reduce research to practice gap | 12 (100) | 35 (97.2) | 33 (97.1) | 32 (97.0) | 1 |
| I have the skills necessary to conduct D&I research | 6 (50.0) | 16 (44.4) | 12 (35.3) | 24 (72.7) | 0.16 |
| I prioritize conducting/supporting D&I research (e.g. through supervision, provision of funding) | 6 (50.0) | 18 (50.0) | 16 (47.1) | 20 (60.6) | 0.71 |
| I have the knowledge required to conduct D&I research | 6 (50.0) | 14 (38.9) | 11 (32.4) | 22 (66.7) | **0.03** |
| I feel confident I could conduct D&I research | 7 (58.3) | 19 (52.8) | 17 (50.0) | 23 (69.7) | 0.38 |
| I have experience supporting others to engage in D&I research (e.g. through supervision, provision of funding) | 4 (33.3) | 10 (27.8) | 12 (35.3) | 24 (72.7) | **0.001** |
| I have experience conducting/being involved in (e.g. as a collaborator) D&I research | 5 (45.5) | 19 (52.8) | 19 (55.9) | 26 (78.8) | 0.07 |
| More often than not, I engage/collaborate with stakeholders and involve them in the design and conduct of my research | 9 (75.0) | 26 (72.2) | 22 (64.7) | 27 (81.8) | 0.47 |
| My research has real-world relevance | 11 (91.7) | 34 (94.4) | 31 (91.2) | 33 (100.0) | 0.35 |
| My research has real-world impact | 10 (90.9) | 31 (86.1) | 27 (79.4) | 32 (97.0) | 0.15 |
| I would like my research to have greater real-world impact | 9 (81.8) | 32 (88.9) | 27 (79.4) | 27 (81.8) | 0.73 |
| D&I science has the potential to improve the real-world impact of my research | 7 (63.6) | 34 (94.4) | 30 (88.2) | 30 (90.9) | 0.06 |
| D&I science is not immediately relevant/applicable to my area of research | 3 (27.3) | 1 (2.8) | 5 (14.7) | 0 (0.0) | **0.004** |
| ***Organisational level factors*** | | | | | |
| My supervisors/colleagues think it is important to conduct D&I research | 7 (58.3) | 29 (80.6) | 22 (64.7) | 23 (69.7) | 0.36 |
| My organisation supports me to conduct/engage in (e.g. as a collaborator) D&I research | 7 (63.6) | 27 (75.0) | 19 (55.9) | 23 (69.7) | 0.39 |
| ***System level factors*** | | | | | |
| Funding agencies in my country more likely to fund D&I research | 4 (36.4) | 18 (50.0) | 13 (38.2) | 17 (51.5) | 0.62 |
| Journals in field are less likely to publish D&I research | 3 (27.3) | 12 (33.3) | 9 (26.5) | 13 (39.4) | 0.72 |

Data reported is a combined score relating to those who stated ‘Agree’ and ‘Strongly agree’. ECR – Early Career Researcher, MCR – Mid-Career Researcher, FTE – Full Time Equivalent, D&I – Dissemination and Implementation. Fisher’s exact tests were used to test for associations between academic career stage and agreement with each of the 17 items (‘perceptions of D&I’); p value significant at <0.05 (bold).
