# Supplementary material for: What hinders and helps academics to conduct Dissemination and Implementation (D&I) research in the field of nutrition and physical activity? An international perspective
Source: Int J Behav Nutr Phys Act. 2020 Jan 16;17:7. doi: 10.1186/s12966-020-0909-z (PMC6966833; doi:10.1186/s12966-020-0909-z)
Supplement: Supplementary file 5 — Additional file 5. Levels of perceived understanding, knowledge, engagement with and importance of D&I by country of work, Table comparing differences in perceptions of D&I by participant country of work. [file 12966_2020_909_MOESM5_ESM.docx]

**Additional File 5. Levels of perceived understanding, knowledge, engagement with and importance of D&I by country of work**

|  | **Country (% agreement)** | | | | | ***P*** |
| --- | --- | --- | --- | --- | --- | --- |
|  | Australia  n (%) | Netherlands n (%) | USA  n (%) | Canada  n (%) | UK  n (%) |  |
| ***Individual level factors*** | | | | | | |
| D&I science is important to reduce research to practice gap | 51 (94.4) | 24 (100.0) | 15 (93.8) | 15 (100) | 12 (92.3) | 0.61 |
| I have the skills necessary to conduct D&I research | 21 (38.9) | 9 (37.5) | 15 (93.8) | 9 (60.0) | 10 (76.9) | **0.001** |
| I prioritize conducting/supporting D&I research (e.g. through supervision, provision of funding) | 22 (40.7) | 9 (37.5) | 14 (87.5) | 8 (53.3) | 8 (61.5) | **0.01** |
| I have the knowledge required to conduct D&I research | 19 (35.2) | 8 (33.3) | 12 (75.0) | 10 (66.7) | 8 (61.5) | **0.01** |
| I feel confident I could conduct D&I research | 23 (42.6) | 11 (45.8) | 14 (87.5) | 10 (66.7) | 9 (69.2) | **0.01** |
| I have experience supporting others to engage in D&I research (e.g. through supervision, provision of funding) | 18 (33.3) | 7 (29.2) | 11 (68.8) | 7 (46.7) | 6 (46.2) | 0.09 |
| I have experience conducting/being involved in (e.g. as a collaborator) D&I research | 27 (50.0) | 12 (52.2) | 15 (93.8) | 11 (73.3) | 8 (61.5) | **0.01** |
| More often than not, I engage/collaborate with stakeholders and involve them in the design and conduct of my research | 30 (55.6) | 20 (83.3) | 15 (93.8) | 14 (93.3) | 11 (84.6) | **0.002** |
| My research has real-world relevance | 51 (94.4) | 22 (91.7) | 16 (100.0) | 15 (100.0) | 13 (100.0) | 0.76 |
| My research has real-world impact | 43 (79.6) | 21 (91.3) | 16 (100.0) | 15 (100.0) | 12 (92.3) | 0.96 |
| I would like my research to have greater real-world impact | 49 (90.7) | 17 (73.9) | 12 (75.0) | 11 (73.3) | 13 (100.0) | **0.05** |
| D&I science has the potential to improve the real-world impact of my research | 46 (85.2) | 21 (91.3) | 14 (87.5) | 14 (93.3) | 13 (100.0) | 0.72 |
| D&I science is not immediately relevant/applicable to my area of research | 3 (5.6) | 2 (8.7) | 1 (6.3) | 1 (6.7) | 2 (15.4) | 0.74 |
| ***Organisational level factors*** | | | | | | |
| My supervisors/colleagues think it is important to conduct D&I research | 40 (74.1) | 17 (70.8) | 9 (56.3) | 10 (66.7) | 8 (61.5) | 0.66 |
| My organisation supports me to conduct/engage in (e.g. as a collaborator) D&I research | 36 (66.7) | 15 (65.2) | 11 (68.8) | 11 (73.3) | 7 (53.9) | 0.87 |
| ***System level factors*** | | | | | | |
| Funding agencies in my country more likely to fund D&I research | 24 (44.4) | 13 (56.5) | 9 (56.3) | 7 (46.7) | 2 (15.4) | 0.15 |
| Journals in field are less likely to publish D&I research | 17 (31.5) | 4 (17.4) | 5 (31.3) | 4 (26.7) | 7 (53.9) | 0.27 |

Data reported is a combined score relating to those who stated ‘Agree’ and ‘Strongly agree’. D&I – Dissemination and Implementation. Fisher’s exact tests were used to test for associations between country of work and agreement with each of the 17 items (‘perceptions of D&I’); p-value significant at <0.05 (bold).
